# Bioluminescent imaging to investigate *Coxiella burnetii* pathogenesis identifies adipose tissue as a host niche for infection

**DOI:** 10.1128/iai.00080-25

**Published:** 2025-06-30

**Authors:** Jenna M. Andrews, Craig R. Roy

**Affiliations:** 1 Department of Microbial Pathogenesis, Yale University School of Medicine 198940 , New Haven, Connecticut, USA; University of Pennsylvania Perelman School of Medicine, Philadelphia, Pennsylvania, USA

**Keywords:** *Coxiella*, Q fever, pathogenesis, *in vivo* imaging

## Abstract

*Coxiella burnetii* is a gram-negative, obligate intracellular pathogen that causes Q fever in humans. *In vivo* research on *C. burnetii* is limited due to the classification of the Nine Mile phase I (NMI) strain as a select agent that requires biosafety level 3 containment. The isogenic Nine Mile phase II (NMII) strain can be cultured safely at biosafety level 2 and has been shown to infect immunocompromised mice, which suggests this strain could be used to investigate virulence phenotypes *in vivo*. This study developed a bioluminescent imaging (BLI) model using NMII to non-invasively monitor *C. burnetii* infections in mice. Here, we show that BLI enables tracking of bacteria in an animal host, identification of bacterial virulence differences, and investigation of host determinants of immunity. Using BLI, we show that NMII resides primarily in visceral adipose tissue following intraperitoneal infection of mice. Intracellular replication of *C. burnetii* in adipocytes was confirmed using cultured cells *ex vivo*. These data indicate that adipose tissue can serve as a niche for *C. burnetii* replication. This study underscores the utility of BLI in advancing *C. burnetii* research and highlights the need for further exploration into the role of adipocytes in the disease Q fever and bacterial persistence *in vivo*.

## INTRODUCTION

*Coxiella burnetii* is a Gram-negative bacterium that typically circulates within livestock populations, but humans may be exposed through inhalation of parturient secretions from infected animals (1, 2). Human disease caused by *C. burnetii*, known as Q fever, generally presents as an acute, flu-like illness; however, a minority of patients develop chronic Q fever, leading to severe complications such as valvular endocarditis, which can be fatal (3, 4). The current treatment for chronic Q fever is a long, aggressive antibiotic regimen known to decrease patient compliance, leading to reactivation of the infection and antibiotic resistance (5, 6).

*Coxiella burnetii* is an obligate intracellular bacterial pathogen that uses a Dot/Icm type IVB secretion system (T4SS) to translocate over 100 different bacterial effector proteins into the host cell cytosol (7, 8). These proteins manipulate and subvert host cell machinery to create and maintain the *Coxiella*-containing vacuole (CCV) and evade host defenses (9, 10). The Dot/Icm system and the collective activity of the effectors are required to promote *C. burnetii* replication intracellularly (10, 11). The study of *C. burnetii* effector proteins has been facilitated by the formulation of axenic media that facilitates genetic manipulation and has led to numerous screens aimed at determining effector functions (10, 12–14).

To date, animal models of disease have not been used to study the role of effectors. This is primarily because studies on *in vivo* pathogenesis of the virulent Nine Mile phase I (NMI) strain of *C. burnetii* are hindered by its status as a category B select agent by the Centers for Disease Control and Prevention (15). The designation of select agent status for certain infectious diseases enhances the protection of both researchers and the general population, but significantly limits the ability to conduct studies on these pathogens. The isogenic Nine Mile phase II clone RSA493 (NMII) is exempt from the select agent classification.

NMII was generated by serial passaging of NMI in embryonated eggs and harbors a chromosomal deletion that eliminates multiple genes involved in lipopolysaccharide (LPS) biosynthesis (16, 17). NMII produces a truncated (rough) LPS lacking the outer core and O-antigen. However, NMII remains capable of infecting host cells and has allowed researchers to study *C. burnetii* pathogenesis in cultured host cells in a biosafety level 2 environment (18). This has broadened access to *Coxiella* research, but largely to the extent of *in vitro* and *ex vivo* work. *In vivo* research remains difficult because NMII is sensitive to fresh serum that facilitates bacterial clearance in most immunocompetent animal models (19). The *in vivo* phenotypes of the NMII strain have garnered increased attention lately. Multiple studies have demonstrated that severe combine immunodeficient (SCID) mice, which lack a fully functioning immune system, show susceptibility to NMII infection (20–22).

To address the need for a more accessible method of detecting pathogenesis phenotypes of effector mutants *in vivo*, we created a bioluminescent imaging (BLI) model using *C. burnetii* NMII. BLI is a technique that employs sensitive cameras to accurately detect light emitted by a pathogen-encoded luminescent protein without invasive procedures. This highly accurate predictive technique enables the identification of infection sites and pathogen prevalence without the need to euthanize animals for comprehensive investigative histopathology and tissue analysis (23). Additionally, BLI provides the unique ability to follow each animal throughout the infection to better track variances in individual responses to infection. Although BLI has been applied to monitor a variety of viral and bacterial infections, this technique has not been used with *C. burnetii*. Here, we demonstrate that BLI can detect Dot/Icm-dependent virulence differences in NMII-infected mice, illustrate the role of complement component C3 in NMII clearance, and demonstrate that NMII accumulates and replicates in adipose tissue after intraperitoneal (IP) infection.

## RESULTS

### Bioluminescence imaging demonstrates the requirement for a functional Dot/Icm system for *C. burnetii* virulence in mice

**Fig 1:**
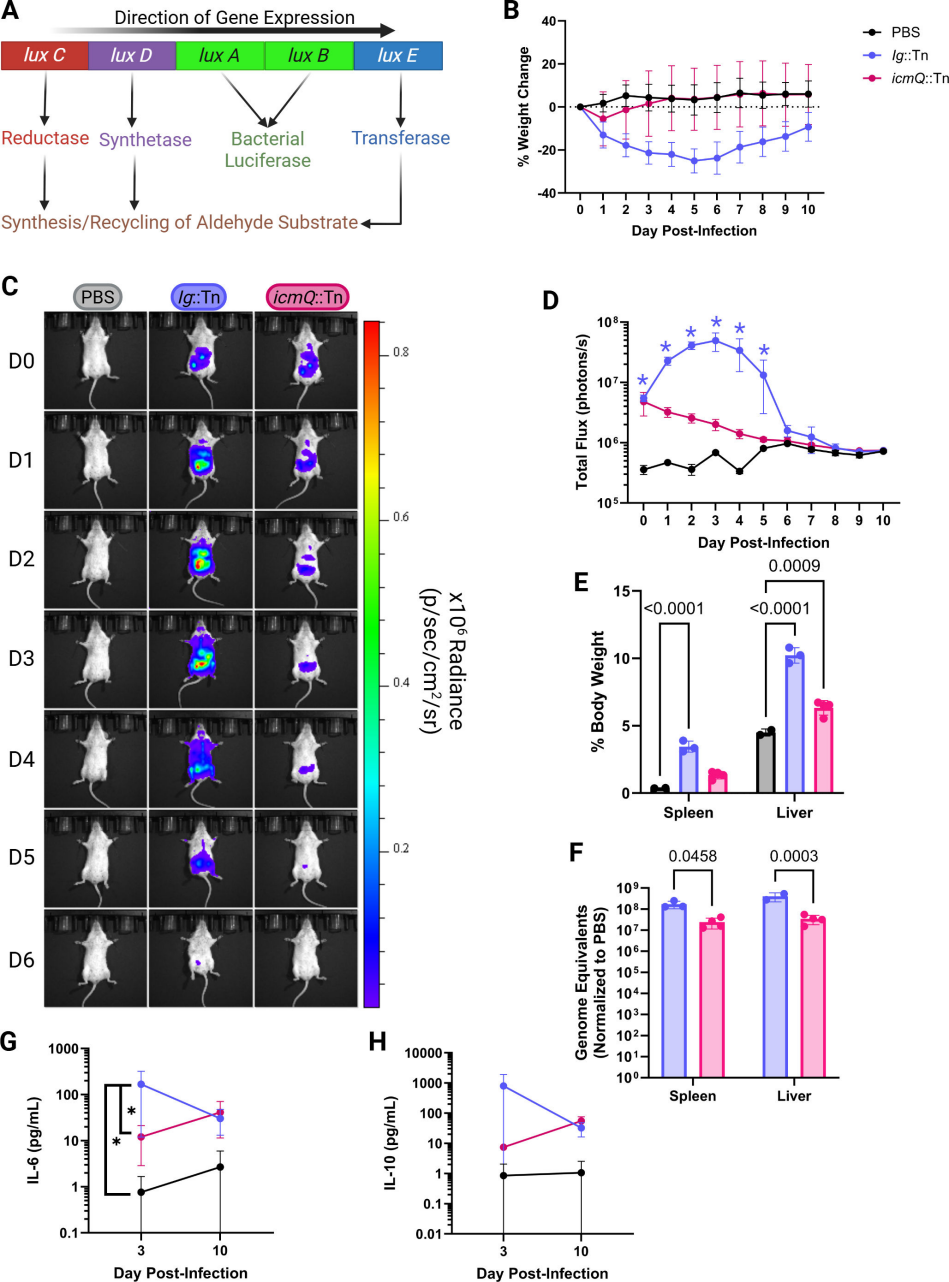
BLI demonstrates a Dot/Icm-dependent infection of mice. (**A**) *lux*CDABE operon from *P. luminescens* (diagram adapted from van Zyl et al.) (**B**) Percent change in body weight of *C. burnetii*-infected A/J mice after intraperitoneal infection with 10^9^ GEs. (**C**) Representative BLI of mice infected with indicated luminescent *C. burnetii* strains at different days post-infection (D0–D6). Radiance color scale: min = 1.86e^4^, max = 1.70e^5^. (**D**) Total flux (photons/s) of *C. burnetii in vivo*. Blue asterisks denote *P*-value <0.05 for Ig::Tn compared to PBS and *icmQ*::Tn. (**E**) Hepatosplenomegaly on day 10 post-infection shown as organ weight as a percentage of total body weight. (**F**) *C. burnetii* GEs on day 10 post-infection in spleens and livers normalized to PBS controls. One mouse in Ig::Tn had undetectable levels of GEs in the liver. (**G**) Serum levels of IL-6 (pg/mL). *P < 0.05. (**H**) Serum levels of IL-10 (pg/mL). All *P*-values are based on two-way analysis of variance and Tukey’s multiple comparison test with single pooled variance; significant *P*-values (*P* < 0.05) are indicated. Data are representative of three technical replicates (PBS *n* = 2) and one biological replicate.

We have previously used a strain of *C. burnetii* NMII expressing the *lux*CDABE operon from *Photorhabdus luminescens* (Fig. 1A) to examine intracellular replication of bacteria in cell culture by measuring bioluminescence (24–27). There was a direct correlation between an increase in bacterial luminescence and an increase of *C. burnetii* genome equivalents (GEs) in a cell culture model, which suggested that bioluminescence could be utilized to assess bacterial replication and dissemination in a mouse model of disease (26). Recently, the attenuated NMII strain has been shown to infect and replicate in SCID mice, experiments we successfully reproduced (Fig. S1); however, the propensity of SCID mice to develop thymic tumors complicated the analysis of *C. burnetii*-specific virulence readouts and necessitated the exploration of alternative mouse models for NMII infections (21, 22, 28). Toward this end, A/J mice, which have been shown to be more susceptible to infection with *C. burnetii*, were infected intraperitoneally with 10^9^ luminescent *C. burnetii* and monitored over 10 days (29–31). The NMII strain used has a neutral transposon insertion in an intergenic (Ig) region of the chromosome (Ig::Tn) and has a wild-type (WT) NMII phenotype (14). An isogenic strain with a transposon insertion in *icmQ*, which encodes for an essential component of the Dot/Icm secretion system, was used as the negative control (*icmQ*::Tn), as this strain does not replicate intracellularly (32). Mice injected with sterile phosphate buffered saline (PBS) served as the uninfected control. We initially tested an intranasal route of infection in mice to mimic aerosol infections in humans; however, the bioluminescent signal obtained by the intranasal method was weak, and detection of bacterial luminescence through multiple tissue layers was not achieved (Fig. S2). Thus, we opted to continue with the intraperitoneal route of infection, a widely accepted approach for conducting *C. burnetii* virulence studies in murine models (21).

In contrast to mice infected with the *icmQ*::Tn strain or the PBS vehicle, mice infected with the Ig::Tn strain exhibited gross symptoms of illness including weight loss over the first 5 days (Fig. 1B), lethargy, scruffy fur, and hunched posture. Additionally, mice infected with the Ig::Tn strain displayed a gradual increase in radiance (photon/s/cm^2^/steradian) (Fig. 1C) and total flux (photons/s) over the first 3 days of infection (Fig. 1D), indicating that this strain was replicating in the mice. Weight differences in Ig::Tn-infected mice demonstrated a correlation with an increase and decrease in bacterial total flux, indicating that as bacterial populations increase, the mice experience greater morbidity, whereas a reduction in luminescence suggests host-mediated clearance. In contrast, both the radiance and total flux measurements exhibited a consistent decline over time in mice infected with the *icmQ*::Tn mutant, indicating that the T4SS-deficient mutant was unable to replicate and was rapidly eliminated by the host immune system (Fig. 1C and D).

It is important to note that for *C. burnetii,* the luminescent signal is primarily detected when the bacteria are in their metabolically active stage displayed by large cell variants (LCVs). The transition of *C. burnetii* from the replicative LCV to the dormant small cell variant (SCV) in axenic medium *in vitro* correlated with a decrease in bacterial luminescence (Fig. S3) (33–35). Thus, although luminescence provides an accurate means of assessing bacterial replication and distribution *in vivo*, a decrease in luminescence does not necessarily mean that bacterial numbers have decreased. Instead, it may indicate a reduction or cessation of metabolic processes within the bacterial population.

Mice were euthanized on day 10 post-infection, and hepatosplenomegaly was observed in the Ig::Tn-infected mice, and to a lesser extent in *icmQ*::Tn-infected mice (Fig. 1E). *C. burnetii* GEs were higher in the organs of Ig::Tn-infected mice compared to those infected with the *icmQ*::Tn mutant (Fig. 1F). The observed increase in hepatosplenomegaly and GEs in mice infected with Ig::Tn, compared to those infected with an *icmQ*::Tn mutant, is consistent with previous studies demonstrating that the *C. burnetii* NMII strain is capable of replication in an immunodeficient mouse model (21, 22). This finding was further corroborated by the observation that the isogenic Dot/Icm-deficient NMII strain displayed reduced hepatosplenomegaly and lower GE counts. Similar results were observed when mice were infected intranasally with luminescent *C. burnetii* (Fig. S2). Blood was collected on days 3 and 10 post-infection and subsequently analyzed for inflammatory cytokines. Levels of interleuken-6 and -10 (IL-6 and IL-10) peaked in the Ig::Tn-infected mice concurrently with bacterial burden on day 3 and decreased by day 10, whereas mice infected with the *icmQ*::Tn mutant exhibited a modest increase in IL-6 and IL-10 levels at day 10 (Fig. 1G and H, respectively).

### BLI reveals that *C. burnetii* accumulates in adipose tissue during infection

**Fig 2:**
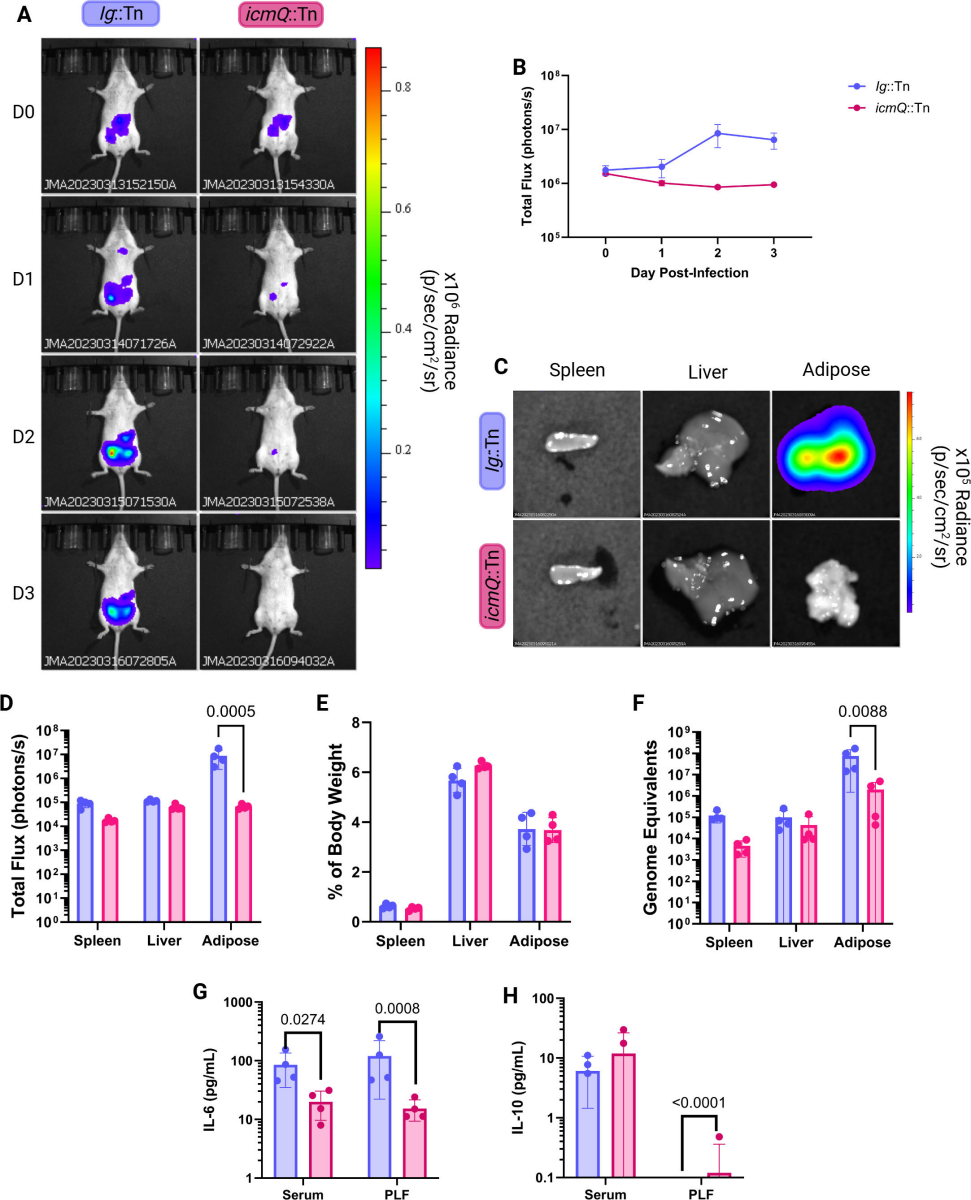
*C. burnetii* localizes to the abdominal adipose tissue in A/J mice. (**A**) Representative BLI of mice infected with indicated luminescent NMII strains at different days post-infection (D0–D3). Radiance color scale: min = 2.11e^4^, max = 9.06e^5^. (**B**) Total flux (photons/s) of infected mice. (**C**) Representative BLI of *ex vivo* organs. Radiance color scale: min = 9.10e^3^, max = 7.86e^5^. (**D**) Total flux (photons/sec) of *ex vivo* organs at day 3 post-infection. (**E**) Organ weight as a percentage of total body weight on day 3 post-infection. (**F**) GEs extracted from organs at day 3 post-infection. (**G**) IL-6 levels (pg/mL) in serum and peritoneal lavage fluid (PLF) at day 3 post-infection. (**H**) IL-10 levels (pg/mL) in serum and PLF at day 3 post-infection. All *P*-values based on unpaired two-tailed *t*-test. All significant *P*-values (*P* < 0.05) are indicated in each panel. Data are representative of four technical replicates and one biological replicate.

To better understand bacterial distribution in mice at peak infection, A/J mice were euthanized on day 3 post-infection, corresponding to the observed peak in bacterial luminescence in mice infected with the Ig::Tn strain (Fig. 2A and B). At this time point, whole body BLI indicated *C. burnetii* luminescence emanating primarily from the lower abdominal area (Fig. S4). BLI of isolated organs from these mice imaged individually revealed a high level of bacterial luminescence in the visceral adipose tissue (Fig. 2C and D), specifically in the gonadal, retroperitoneal, and mesenteric depots (36).

Hepatosplenomegaly did not differ among infection groups at day 3 post-infection (Fig. 2E); however, GE measurements confirmed increased replication of the Ig::Tn strain compared to the *icmQ*::Tn mutant (Fig. 2F). In addition to measuring serum cytokines, we analyzed the local immune response by analyzing the peritoneal lavage fluid (PLF). Compared to mice infected with the *icmQ*::Tn mutant, mice infected with the Ig::Tn strain had approximately 10-fold higher IL-6 levels in sera and PLF at day 3 post-infection (Fig. 2G). IL-10 levels in PLF were similar across infection groups (Fig. 2H).

Our BLI data demonstrating that *C. burnetii* can localize and replicate in adipose tissue are consistent with previous research showing that *C. burnetii* phase I genomic DNA could be detected in various adipose tissues in BALB/c and C57BL/6 mice 4 months after the infection had presumably cleared (37). Bacterial persistence has not been investigated in depth for *C. burnetii*, but adipose tissue has been implicated in the persistence of other bacterial pathogens such as *Mycobacterium tuberculosis*, *Chlamydia pneumoniae*, and *Staphylococcus aureus* (3, 37–41). We hypothesized that *C. burnetii* could remain dormant in the adipose tissue long after the infection has resolved, and immunosuppression could lead to the reactivation of infection (42). We performed an experiment attempting to reactivate our NMII infections in mice using dexamethasone-mediated immunosuppression of mice that had been allowed to clear and recover from an initial infection but found no evidence of NMII reactivation under these conditions (Fig. S5), which differs from previous studies showing reactivation of NMI under similar infection conditions (19, 42, 43).

### BLI demonstrates a role for the complement component C3 in host defense to *C. burnetii*

Having established that BLI can detect Dot/Icm-dependent virulence phenotypes in murine models, we next investigated whether BLI could be used to assess host factors important for defense during *C. burnetii* infection. Previous *in vitro* data have shown *C. burnetii* NMII is sensitive to host serum, which correlates with the activation of the alternative complement pathway requiring the complement component C3 (19). Thus, we sought to investigate the role of C3 in resolving infection by *C. burnetii* NMII using the C3 knockout mouse model B6.129S4-C3^tm1Crr^/J (C3 KO).

**Fig 3:**
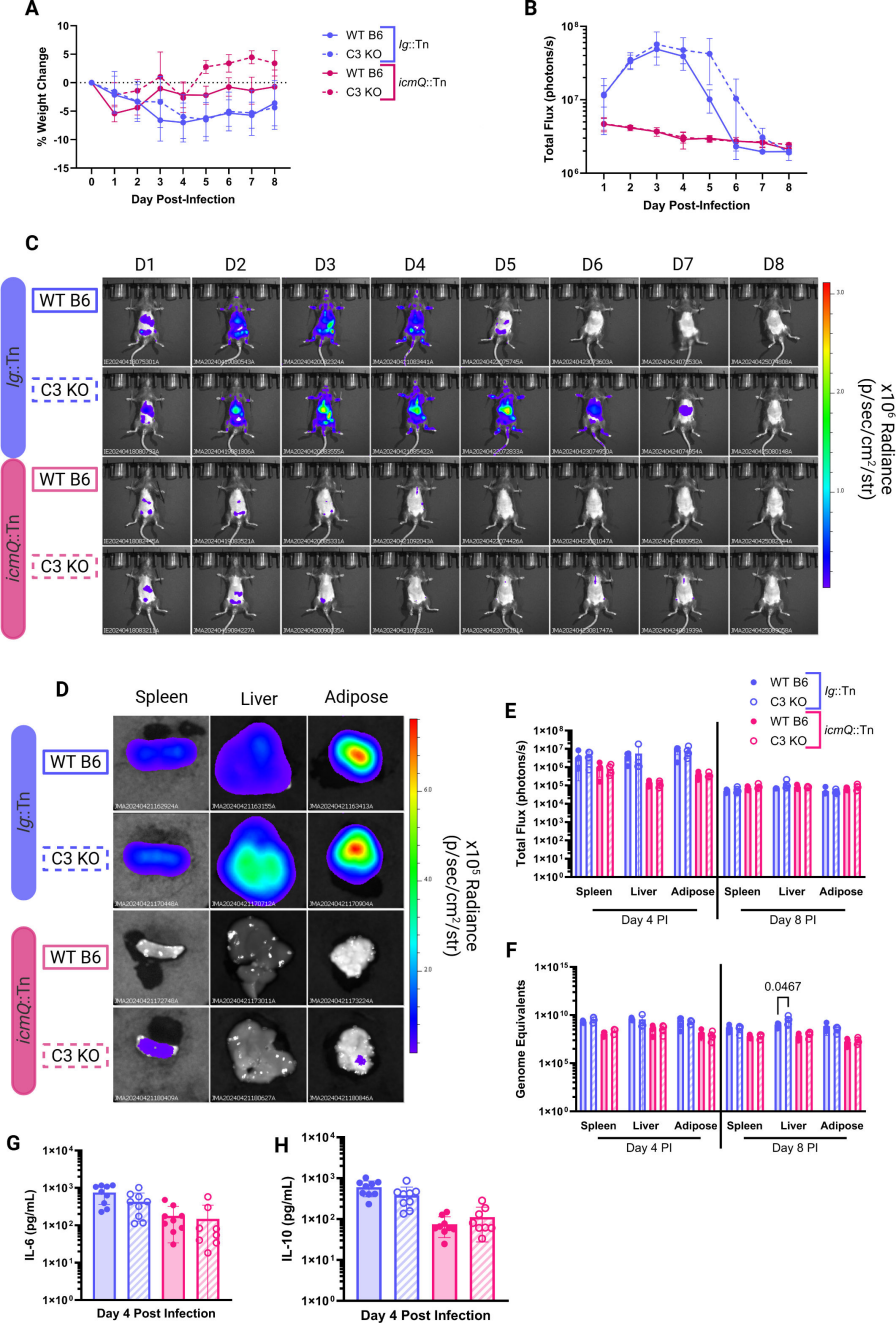
BLI demonstrates a role for complement component C3 in clearing *C. burnetii* infections. (**A**) Percent change in body weight of NMII-infected C57BL/6J (WT B6) and B6.129S4-C3^tm1Crr^/J (C3 KO) mice. (**B**) Total flux (photons/s) of *C. burnetii in vivo*. (**C**) Representative BLI of infected mice infected with indicated luminescent NMII strains at different days post-infection (D1–D8). Radiance color scale: min = 3.46e^4^, max = 3.12e^6^. (**D**) Representative BLI of organs on days 4 and 8 post-infection. Radiance color scale: min = 1.69e^4^, max = 7.62e^5^. (**E**) Total flux (photons/s) of organs on day 6 post-infection. *P*-values based on two-way analysis of variance (ANOVA) and Tukey’s multiple comparisons test with a single pooled variance are indicated. (**F**) *C. burnetii* GEs in organs on days 4 and 8 post-infection. *P*-values based on two-way ANOVA and Tukey’s multiple comparisons test with a single pooled variance. (**G**) Serum levels of IL-6 (pg/mL) on day 4 post-infection. *P*-values based on one-way ANOVA and Tukey’s multiple comparisons test with a single pooled variance are indicated. (**H**) Serum IL-10 (pg/mL) on day 4 post-infection. *P*-values based on one-way ANOVA and Tukey’s multiple comparisons test with a single pooled variance. All significant *P*-values (*P* < 0.05) are indicated in each panel. Data are representative of four technical replicates and one biological replicate; cytokine levels in G and H include data for all mice at day 4, thus *n* = 8.

C57BL/6J (WT B6) and congenic C3 KO mice were infected intraperitoneally with either the luminescent Ig::Tn or the *icmQ*::Tn strain of NMII. Both the WT B6 and C3 KO mice infected with Ig::Tn displayed increased weight loss compared to *icmQ*::Tn-infected mice (Fig. 3A). Elevated levels of total flux (Fig. 3B) and radiance (Fig. 3C) were observed through day 6 post-infection in WT B6 mice and through day 8 in C3 KO mice infected with the Ig::Tn strain, suggesting that C3 KO mice exhibited delayed clearance of infection compared to WT B6 mice. However, the C3 KO mice displayed mild symptoms of infection in contrast to the WT B6 mice, which displayed more exaggerated scruffiness, lethargy, and hunched posture. Both mouse strains cleared the *icmQ*::Tn mutant rapidly, with no significant differences observed in the rate of bacterial clearance (Fig. 3B and C), suggesting that C3 function does not play a role in the elimination of infection caused by a strain deficient in intracellular replication. Mice were euthanized at day 4 and day 8 post-infection, and isolated organs were analyzed *ex vivo* for bacterial luminescence. The radiance, flux, and GEs observed in WT B6 and C3 KO mice were comparable across all organs (Fig. 3D through F, respectively) at the peak of infection (day 3) and subsequently as the infection approached clearance (day 8). Thus, although the Ig::Tn strain was cleared more rapidly in the WT B6 mice compared to the C3 KO mice, this did not correlate with increased bacterial loads in these tissues.

Analyses of sera demonstrated elevated levels of the cytokines IL-6 and IL-10 in the in wild-type B6 mice compared to C3 knockout mice following infection with the Ig::Tn strain (Fig. 3G and H, respectively). These data suggest that the presence of a functional complement system in WT B6 mice likely contributed to an enhanced inflammatory response to *C. burnetii* infection, leading to heightened cytokine production. These data potentially explained the exacerbated disease manifestations observed in WT B6 mice. Both mouse strains exhibited low levels of IL-6 and IL-10 following infection with the *icmQ*::Tn mutant, which is consistent with the rapid clearance of this mutant by the immune system. Overall, these data demonstrate the efficacy of BLI in examining host determinants of pathogenesis and indicate that C3 contributes to, but is not essential for, the clearance of an NMII infection *in vivo*.

**Fig 4:**
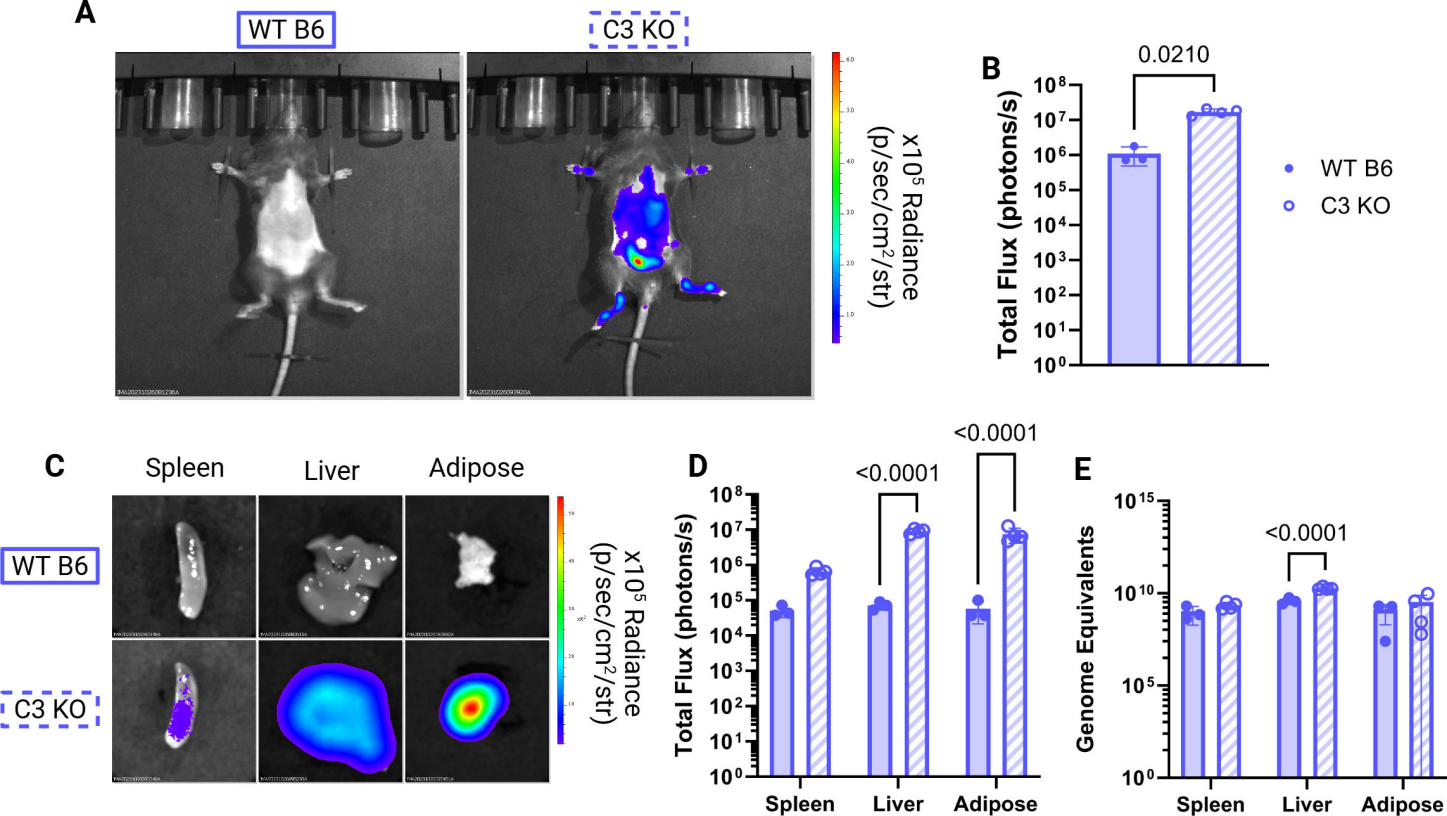
BLI can detect differences in bacterial burden in isolated tissue. (**A**) Representative BLI of WT B6 and C3 KO mice at day 6 post-infection with the Ig::Tn strain. Radiance color scale: min = 4.58e^4^, max = 6.18e^5^. (**B**) Total flux (photons/s) of mice at day 6 post infection. *P*-values based on paired *t*-test are indicated. (**C**) Representative BLI of gross organs at day 6 post-infection. Radiance color scale: min = 1.84e^4^, max = 5.38e^5^. (**D**) Total flux (photons/s) of *ex vivo* organs at day 6 post-infection. *P*-values based on two-way analysis of variance (ANOVA) and Sidak’s multiple comparisons test with a single pooled variance are indicated. (**E**) *C. burnetii* GEs extracted from organs at day 6 post-infection. *P*-values based on two-way ANOVA with uncorrected Fisher’s least significant difference (LSD) with a single pooled variance are indicated. All significant *P*-values (*P* < 0.05) are indicated. Data representative of three to four technical replicates and one biological replicate.

Because bacterial luminescence was lower in the whole animal for WT B6 mice compared to the C3 KO mice at day 6 post-infection with the Ig::Tn strain, we decided to investigate whether this difference could be seen in isolated organs by BLI or by quantifying GEs. Consistent with previous assays, total flux measurements taken using whole animal BLI showed higher bacterial luminescence in the C3 KO mice at day 6 compared to the WT B6 mice (Fig. 4A and B). This enhanced bacterial luminescence was also detected in isolated spleen, liver, and adipose tissue of C3 KO mice, but not the WT B6 mice (Fig. 4C). Flux measurements confirmed higher levels of bacterial luminescence in these tissues in C3 KO mice compared to the WT B6 mice (Fig. 4D). Importantly, these differences were not reflected in GE measurements in isolated organs (Fig. 4E). This points to luminescence being more accurate at determining metabolically active or replicating bacteria compared to GEs. Notably, the liver presents with differing GEs between groups, which could indicate that the liver is more efficient in clearing DNA from non-viable NMII or that more distal organs do not accumulate genomic DNA from non-viable bacteria in the initial inoculum. Regardless, these data demonstrate the capacity of BLI to discern bacterial viability and metabolic activity during infection under conditions where bacterial numbers remain comparable.

### *C. burnetii* replicates in adipocytes *in vitro*

**Fig 5:**
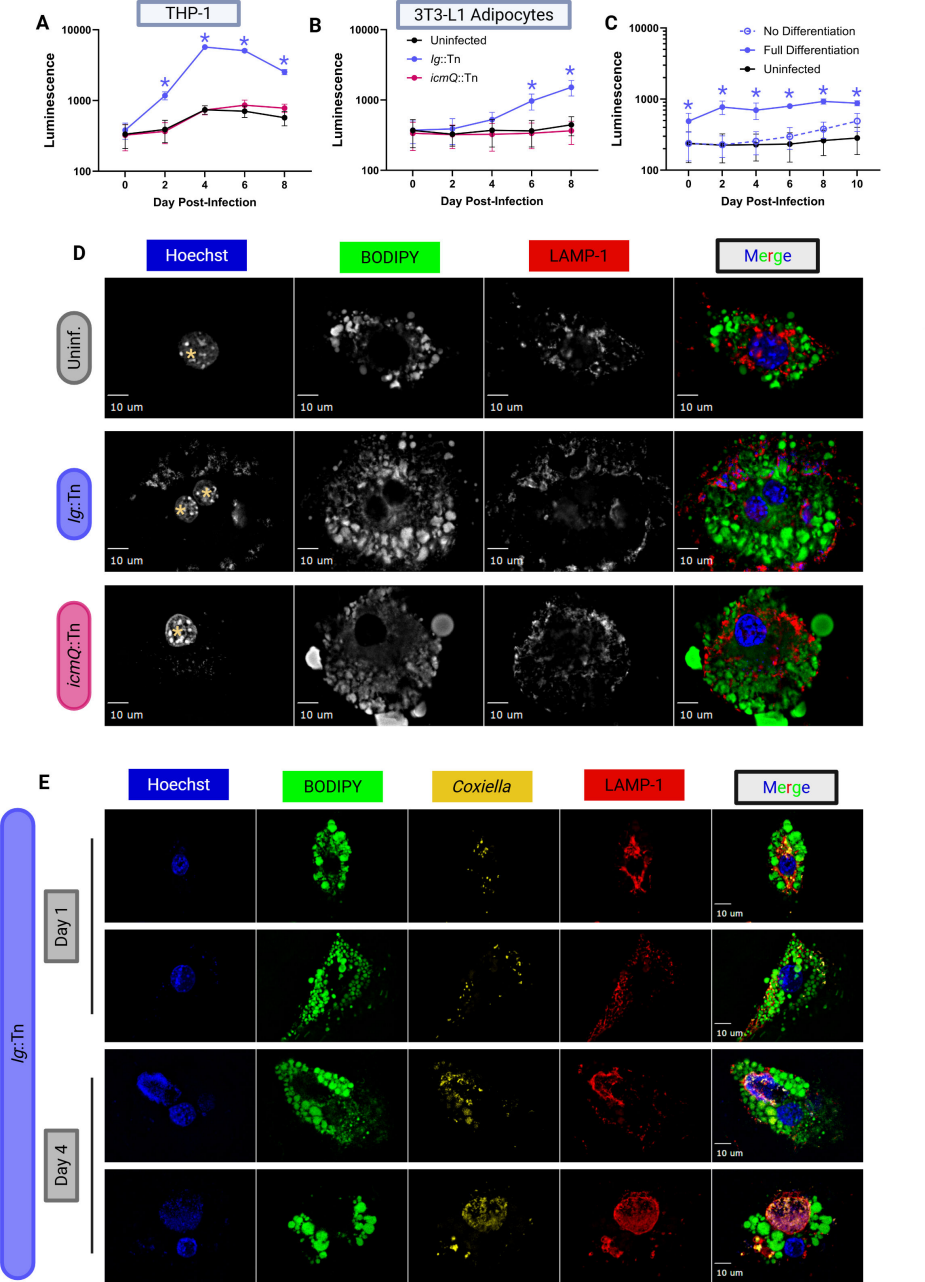
Dot/Icm-dependent replication of *C. burnetii* in cultured adipocytes. (**A**) Intracellular replication of indicated *C. burnetii* strains in THP-1 macrophages and (**B**) in 3T3-L1 adipocytes. (**C**) Intracellular replication of Ig::Tn *C. burnetii* in 3T3-L1 adipocytes and undifferentiated 3T3-L1 cells. (**D**) Representative fluorescence microscopy micrographs illustrating the infection of 3T3-L1 adipocytes with indicated NMII strains and colocalization of *C. burnetii* with LAMP-1 in BODIPY-positive cells (adipocytes) on day 4 post-infection. Yellow asterisks denote cell nuclei. (**E**) Representative fluorescence microscopy micrographs illustrating intracellular replication of Ig::Tn in 3T3-L1 adipocytes at days 1 and 4 post-infection. All *P*-values based on two-way analysis of variance and Tukey’s multiple comparisons test with single pooled variance; significant *P*-values (*P* < 0.05) are indicated for Ig::Tn (**A and B**) and fully differentiated (**C**) with blue asterisk. Data are representative of two technical replicates and three biological replicates.

During our analysis of isolated tissue and organs, it became apparent that one of the major sites of *C. burnetii* luminescence was adipose tissue. Bechah et al. also reported that NMI was capable of replicating in 3T3-L1 adipocytes *in vitro* (37). Collectively, these data indicate that adipose tissue may function as a site for *C. burnetii* replication in animals and serve as a reservoir for non-replicating but viable bacteria. To explore this further, we next examined the ability of the *C. burnetii* NMII strain to replicate in adipocytes *in vitro* using BLI. We had previously used Phorbol 12-myristate 13-acetate (PMA)-differentiated Tohoku Hospital Pediatrics (THP)-1 macrophages to demonstrate that intracellular replication of *C. burnetii* can be measured using bacterial luminescence (26). We utilized our established luminescence assay to evaluate intracellular replication of the Ig::Tn and *icmQ*::Tn strains in 3T3-L1 adipocytes *in vitro. C. burnetii* displayed a Dot/Icm-dependent replication in cultured 3T3-L1 adipocytes (Fig. 5A and B). Upon closer examination of cultured 3T3-L1 adipocytes, we observed a heterogeneous population of cells comprised of fully differentiated adipocytes as well as undifferentiated 3T3-L1 cells. Consequently, we conducted a comparative analysis of NMII replication in cell cultures that had undergone treatment to generate 3T3-L1 adipocytes and cultures containing exclusively undifferentiated 3T3-L1 cells (Fig. 5C). Our data revealed an enhanced replication of NMII in the differentiated adipocytes compared to the undifferentiated 3T3-L1 cells, which suggests adipocytes can support robust replication of *C. burnetii*.

Fluorescence microscopy was used to validate *C. burnetii* replication in adipocytes. Adipocytes were identified in the differentiated 3T3-L1 cells by the presence of BODIPY-stained lipid droplets. Vacuoles containing replicating NMII bacteria were found in large lysosomal associated membrane protein (LAMP)-1-positive vacuoles in the 3T3-L1 adipocytes. By comparison, the T4SS-deficient *icmQ*::Tn mutant was unable to form vacuoles in these cells as demonstrated by the presence of only bacterial puncta and a diffuse LAMP-1 signal (Fig. 5D). Replication of the Ig::Tn in 3T3-L1 adipocytes was further validated by comparing infected cells at days 1 and 4 post-infection using fluorescence microscopy. Large LAMP-1 positive CCVs were only present at day 4 post-infection, but not at day 1 post-infection (Fig. 5E). In addition, *C. burnetii* has been implicated in the T4SS-dependent manipulation of lipid droplet (LD) accumulations and other aspects of cellular lipid metabolism in non-adipocyte cells, and intracellular replication is significantly affected when LD homeostasis is altered, a finding we reproduced in 3T3-L1 adipocytes by blocking lipolysis using atglistatin (Fig. S6) (44, 45). Together, these data indicate that adipocytes support the replication of NMII *in vitro*.

### Adipocytes are the primary cell type supporting *C. burnetii* replication in adipose tissue

**Fig 6:**
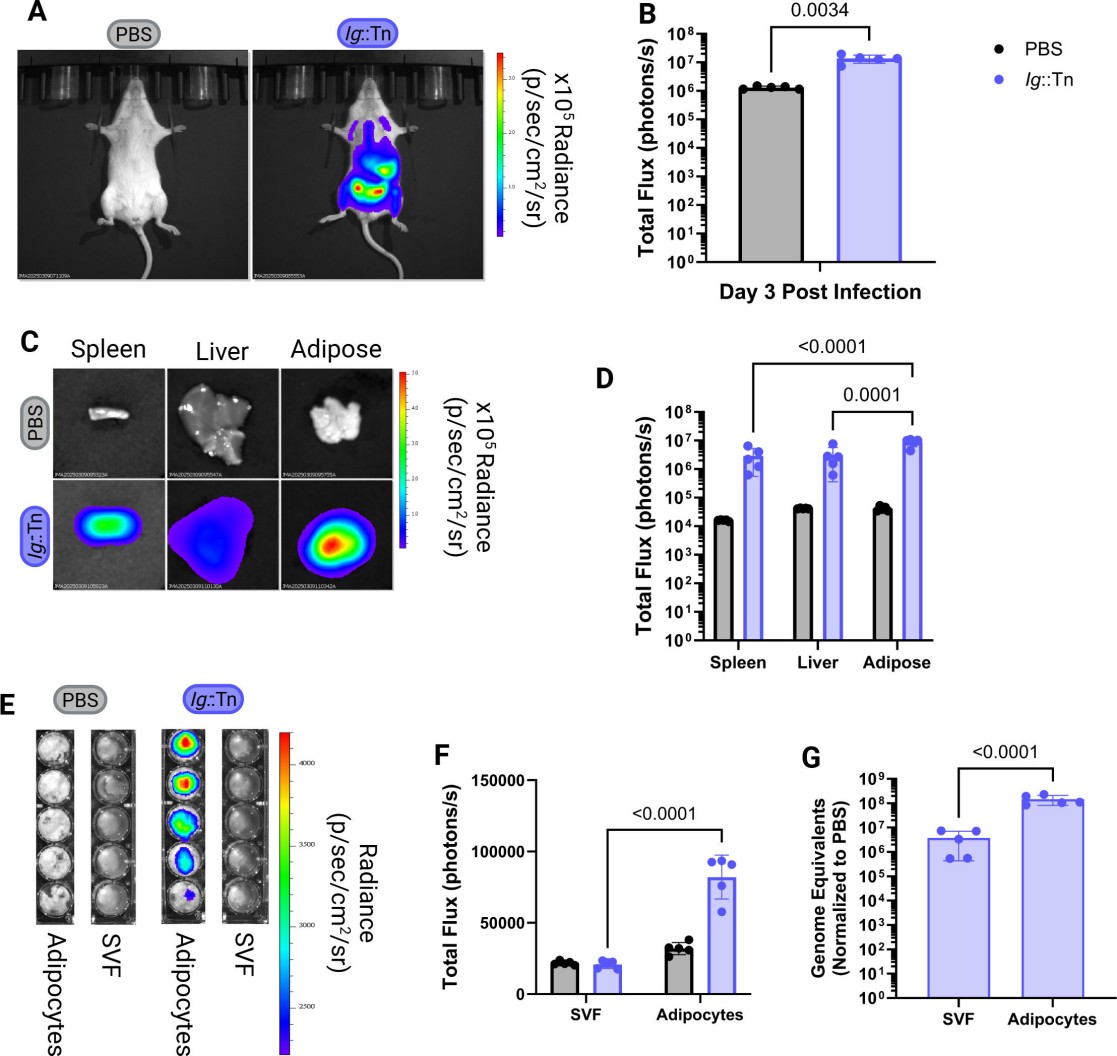
Adipocytes are the primary target of *C. burnetii* in adipose tissue *in vivo*. (**A**) Representative BLI of A/J mice at day 3 post-infection with either the Ig::Tn strain or PBS. Radiance color scale: min = 1.02e^4^, max = 3.49e^5^. (**B**) Total flux (photons/s) of mice at day 3 post-infection. *P*-values based on paired *t*-test are indicated. (**C**) Representative BLI of gross organs at day 3 post-infection. Radiance color scale: min = 6.47e^3^, max = 5.06e^5^. (**D**) Total flux (photons/sec) of *ex vivo* organs at day 3 post-infection. *P*-values based on two-way analysis of variance (ANOVA) and Tukey’s multiple comparisons test with a single pooled variance are indicated. (**E**) Luminescence of the adipocyte fraction and SVF at day 3 post-infection. Radiance color scale: min = 2.21e^3^, max = 4.20e^3^. (**F**) Total flux of the adipocyte fraction and SVF at day 3 post-infection. *P*-values based on two-way ANOVA and Sidak’s multiple comparisons test with a single pooled variance are indicated. (**G**) *C. burnetii* GEs extracted from adipocytes and SVF at day 3 post-infection. *P*-values based on two-way ANOVA and Sidak’s multiple comparisons test with a single pooled variance are indicated. All significant *P*-values (*P* < 0.05) are indicated. Data are representative of five technical replicates and one biological replicate.

To better understand what cells in adipose tissue were supporting *C. burnetii* replication, we removed the tissue from A/J mice at day 3 post-infection (Fig. 6A through D), created a single cell suspension, and separated the adipocytes from the other cell types using centrifugation. In this differential centrifugation protocol, the more buoyant adipocytes are separated from cells in the pelleted stromal vascular fraction (SVF). When adipose tissue fractions were imaged *ex vivo*, fluorescence was detected only in the adipocyte cell fraction from Ig::Tn-infected mice (Fig. 6E and F). No luminescence was detected for cells in the SVF fraction from Ig::Tn-infected mice when compared to the negative controls, which were cell fractions collected from mice receiving only PBS. Additionally, *C. burnetii* GE counts were significantly higher in the adipocyte cell fraction from Ig::Tn-infected mice compared to the SVF fraction (Fig. 6G). Together, these data indicate that adipocytes represent a major reservoir for *C. burnetii* replication during infection *in vivo*.

## DISCUSSION

Here, we present BLI as an approach for temporally monitoring a *C. burnetii* infection *in vivo* by providing a way to non-invasively assess bacterial burdens and infection dispersal *in vivo* and target organs for analysis *ex vivo*. Using an *in vivo* imaging system (IVIS), we could detect *C. burnetii* constitutively expressing the *luxCDABE* operon from *P. luminescens in vivo*. Most studies investigating *C. burnetii* pathogenesis *in vivo* use the virulent NMI, limiting work to facilities with biosafety level 3 and select agent authorization (46). More recently, the attenuated NMII strain that produces a truncated LPS has been shown to infect and replicate in SCID mice (21, 22). We have successfully reproduced and demonstrated the efficacy of the SCID mouse model for investigating *C. burnetii* infection, and we additionally show that both A/J and C57BL/6J mice can be used for intraperitoneal NMII infections. In this study, we have presented a variety of uses for BLI, including discerning virulence differences between *C. burnetii* mutant strains, assessing host factors that influence disease progression, and identifying adipose tissue and adipocytes as a niche that supports replication of NMII.

The external presentation of the mice, most hunched/scruffy at day 3 and healthy by day 6 post-infection, correlated with bacterial luminescence, further indicating bacterial expansion and clearance, respectively. All mice infected with the *icmQ*::Tn mutant rapidly cleared the infection, underscoring the importance of a functional Dot/Icm T4SS in establishing an infection. GEs were incompletely correlated with luminescence and appeared to be a lagging indicator on the state of infection, likely because the immune system needs time to fully clear the genomic DNA of dead bacteria. In addition to GEs, hepatosplenomegaly is used as an indicator of disease severity, with larger organ sizes correlating with increased pathogen burdens (due to increased blood flow and immune infiltrates). However, we found this is not necessarily accurate for earlier time points where organ size was indistinguishable between infection groups. Thus, like GEs, organ size appears to be a lagging indicator of virulence. The limitations of GEs and organ size measurements in evaluating *C. burnetii* virulence and tissue infiltration are emphasized by this finding, indicating that BLI has many advantages over other approaches for longitudinal monitoring of infection.

Having determined that BLI could assess bacterial determinants of virulence, we sought to investigate host determinants of pathogenesis using BLI. The truncated LPS structure of NMII is directly bound by complement component C3 and activates the alternative complement pathway (19). We therefore utilized a C3 KO mouse model to determine how the loss of C3 affects NMII clearance *in vivo*. C3 KO mice had similar bacterial burdens compared to WT B6 mice and had delayed clearance of an NMII infection, indicating that C3 is not necessary but contributes to bacterial clearance. This is consistent with previous research showing that NMII is detected by other pattern recognition receptors that remain functional in the C3 KO mice and can activate the inflammatory response through additional pathways (47). C3 KO mice did not show symptoms of illness throughout infection, whereas the WT B6 mice, like the A/J mice, appeared hunched and scruffy until the infection cleared. This dampened immune response was corroborated in the serum levels of the cytokines IL-6 and IL-10 measured in both mouse strains, with C3 KO mice producing less of both cytokines at peak infection (day 3 post-infection) with Ig::Tn compared to WT B6 mice. We postulate that this lack of outward illness in C3 KO mice is due to the reduced level of immune activation resulting from the loss of C3, which is known to be a potent activator of the innate immune response (48).

In addition to *in vivo* imaging, BLI can be utilized *ex vivo* to illuminate specific organs or tissues that may serve as replicative niches for bacteria. When imaged individually, it became evident that NMII bacteria resided primarily in the visceral adipose tissue, and not the spleen or liver, which was unexpected. A previous study found NMI genomic DNA in adipose depots throughout the body of mice and detected it up to 4 months after purported infection resolution (37). Additionally, NMI could infect and replicate in 3T3-L1 adipocytes, a finding we reproduced using NMII. *C. burnetii* can infect a wide range of cell types, and adipose tissue is composed of a variety of cells beyond adipocytes including preadipocytes, fibroblasts, endothelial cells, and various immune cells that are in the SVF. Within the adipose tissue, adipocytes account for less than 50% of the total cell number, but over 90% of the total cell volume due to their large size (47, 49). Undifferentiated 3T3-L1 cells supported lower levels of *C. burnetii* replication than adipocytes, suggesting that the adipocyte form is more susceptible to infection by *C. burnetii*. Consistent with data using cultured adipocytes, we found that after isolation of adipose tissue from mice infected with *C. burnetii*, the bacteria were primarily found in the adipocytes and not in the cells from the SVF. This indicates that adipocytes are physiologically important cell types capable of supporting *C. burnetii* replication during infection.

The interactions between pathogens and adipocytes are an evolving field, and it has emerged that adipose tissue plays a myriad of roles during infections with bacteria, viruses, and parasites (50). Although adipocytes serve as the primary cell type for lipid storage, they are not the only cells with LDs, which are lipid storage organelles important for the replication of multiple pathogens (51). Intracellular bacterial pathogens such as *Salmonella typhimurium, Orientia tsutsugamushi*, *Chlamydia* spp., and *Mycobacterium* spp. have all been found to manipulate host cell LD formation (52–58). *Mycobacterium tuberculosis* has been found in adipose tissue in both infected mice and humans, and *in vitro* infects adipocytes, where it can persist in a non-replicating state (38, 39). *Chlamydophila pneumoniae*, while not yet observed in adipose tissue *in vivo*, replicates in adipocytes *in vitro* by inducing lipolysis, ostensibly to gain energy for its own replication (40). Similarly, *Staphylococcus aureus* infects adipocytes, inhibits lipolysis, and increases monocyte chemoattractant protein (MCP)-1 and IL-6 levels, but there appears to be no change in adipocyte viability, and researchers hypothesize the bacteria could be using the adipose tissue as a reservoir for persistence (41). *C. burnetii* infection is known to affect LD formation, and intracellular replication is significantly affected when LD homeostasis is altered, a finding we replicated in 3T3-L1 adipocytes (44, 45). In adipocytes, *C. burnetii* may not need to direct host cell LD formation, allowing it to allocate resources elsewhere and leading to robust proliferation in adipose tissue *in vivo*. However, further experimental investigation is necessary to evaluate this hypothesis. Although several intracellular bacterial pathogens use adipose tissue as a persistence reservoir, our experiment investigating NMII persistence in adipose tissue was inconclusive. However, we routinely noted an increase in serum IL-6 and IL-10 in both the A/J and WT B6 mice at peak infection with the Ig::Tn NMII strain, which could potentially be related to infection of adipocytes.

Generally, IL-6 is a cytokine elevated during the acute phase of infection acting as a strong activator of the inflammatory response and is also an adipokine (cytokine secreted by adipose tissue) that will induce lipolysis (45). Similarly, IL-10 is a cytokine and adipokine that broadly moderates the inflammatory response and specifically alters adipocyte chromatin structure, repressing thermogenic genes that regulate lipolysis (59, 60). Serum IL-6 levels are elevated during acute *C. burnetii* infection and in Q fever fatigue syndrome patients (61, 62). IL-10 overproduction is seen in the serum of chronic Q fever, where patients present with persistent focalized infections that are more severe than the initial acute infection (3). Mice overproducing IL-10 in the myeloid compartment have a shift in macrophage polarization from the microbicidal M1 to the immunosuppressive M2 phenotype, which may facilitate microbial persistence (3). Knocking out IL-10 reduced bacterial replication in mice, suggesting the bacteria are better contained when the inflammatory response is not modulated by IL-10 (63). Furthermore, ruminant animals are the main host of *C. burnetii*, but camels have shown higher levels of GEs compared to other ruminants in their regions, which could be due to the increased levels of adipose tissue found in camel humps (37, 64). To our knowledge, no adipose tissue samples have been obtained from human patients of chronic Q fever nor in animals with coxiellosis. Further research is necessary to elucidate whether adipose tissue is targeted in human infections and if it serves as a reservoir for chronic Q fever. It remains to be seen whether there is a link between increased IL-6 and IL-10 levels, lipolysis, and *C. burnetii* lipid utilization and bacterial replication, but taken together, bacterial infection of adipocytes has been shown to cause changes to adipocyte physiology that, in some cases, could favor pathogen replication and persistence. Overall, our experiments provide additional evidence that *C. burnetii* may persist in the adipose tissue of Q fever patients.

*In vivo* disease presentation (including bacterial dissemination, tissue infiltration, and cytokine responses) of NMII will differ compared to infection by NMI due to lower levels of replication and enhanced clearance of NMII by the host immune response. Dissemination kinetics may also vary between NMI and NMII when using different mouse strains and mutants. Nonetheless, our results demonstrating adipose infiltration by NMII are consistent with previous reports finding NMI GEs in adipose depots (37), which indicates there are *in vivo* virulence phenotypes that are common to both NMI and NMII. Additionally, the kinetics of *C. burnetii* dissemination by the IP route will be different than when using an aerosol model of infection, which is how most humans become infected. In an aerosol model, *C. burnetii* will first infect alveolar macrophages and then disseminate from the lungs before infecting more distal anatomic sites, which would include lymphoid organs and adipose tissue. Thus, the IP model is more relevant for investigating later stages of *C. burnetii* spread in the host. We examined the mice after intranasal infection using NMII; however, the bioluminescence signal was difficult to detect *in vivo,* which could be due to a combination of factors such as reduced replication in alveolar macrophages and needing increased tissue penetration to detect a luminescence signal emitted by bacteria in the lung. Importantly, these studies provide a template and proof of principle for using BLI to study *C. burnetii* virulence.

In summary, these data indicate that BLI provides numerous advantages over traditional methods for temporally monitoring *C. burnetii* pathogenesis. Fewer animals are required for experiments, imaging is non-invasive, and researchers can track individual animals over time. Additionally, BLI can monitor and quantify metabolically active *C. burnetii in vivo* and provide a readout for virulence that is more difficult to obtain by measuring GEs or organ size. Thus, the use of BLI in the future should aid in determining the role of *C. burnetii* effector proteins in modulating host responses to promote pathogenesis and could be applied to research using fully virulent strains of *C. burnetii*.

## MATERIALS AND METHODS

### Bacterial strains and host cell lines

*C. burnetii* Nine Mile phase II (RSA493) transposon mutant generation was described previously (9, 10, 14, 32) and selected for using 3 µg/mL chloramphenicol. All *C. burnetii* strains were grown axenically in acidified citrate cysteine media (ACCM)-2 media at 5% CO_2_, 2.5% O_2_, at 37°C as previously described (65, 66). No *C. burnetii* strain was passaged more than two times prior to cell or animal infections. Generation of *C. burnetii* strains expressing the *lux*CDABE operon was done using the insertional plasmid pMiniTn7T-Kan-311P-*lux*CDABE-T0T1, constructed and provided by S. Reed (Quinnipiac University) (27). All *C. burnetii* strains were selected using kanamycin (375 µg/mL) and chloramphenicol (3 µg/mL) when appropriate. All GEs were quantified for the *dot*A gene (F: 5′-GCGCAATACGCTCAATCACA-3′, R: 5′-CCATGGCCCCAATTCTCTT-3′) against a standard curve using RT-qPCR. To quantify *scvA* transcripts, intergenic *C. burnetii* cultures were grown in ACCM-D, cultures were diluted to OD_600_ 1.5 for all luminescence readings, and RNA was extracted using the Qiagen RNeasy mini kit, cDNA generated using iScript Adv cDNA kit for RT-qPCR (BioRad 1725038); qPCR for the *scvA* gene (F: 5′-TGGAAAGACAAAATGTCCAACAA-3′, R: 5′-GGTTAGAAGCACCCGGTCGT-3′) (34); groEL (F: 5′-GCGCAGCCGTCGAAGA-3′, R: 5’- CGATTTAAGCACGCGGATTAA-3′) (67). All qPCR reactions were done using iQ SYBR Green Supermix (BioRad 170-8882).

THP-1 cells were grown in Roswell Park Memorial Institute (RPMI) 1640 (ATCC Modification) (Gibco) and supplemented with 10% heat inactivated fetal bovine serum (HI FBS) (% vol/vol); 162 nM PMA (Sigma P1585) for 24 h was used for macrophage differentiation. 3T3-L1 cells were provided by Matthew Rodeheffer at Yale University and grown in high-glucose Dulbecco’s modified Eagle’s medium (DMEM) (Gibco) supplemented with 10% heat-inactivated fetal bovine serum (% vol/vol). Cells were not allowed to exceed 70% confluence. Differentiation into adipocytes was performed as follows: ~35,000 cells per well were plated in a 24-well plate in DMEM supplemented with 3% HI FBS (% vol/vol); cells were allowed to reach confluence and remain at confluence for 2 days; differentiation media was added for 6 days total (refreshed every 3 days) and was composed of 1 mM dexamethasone (Sigma D8893), 5 µg/mL insulin (Sigma I1882), 0.5 mM IBMX (Sigma I5879), and 2 µM rosiglitazone (Sigma R2408) in DMEM + 3% HI FBS; differentiation media was replaced with insulin-only media for 6 days to allow cells to fully differentiate into adipocytes. All cells were maintained in 37°C humidified air at 5% CO_2_. THP-1 cells were passaged no more than 20 times prior to use, and 3T3-L1 cells were passaged no more than 10 times prior to use.

### *C. burnetii* growth curves

THP-1 cells were seeded at a density of 2 × 10^5^ cells per well in a 24-well plate and differentiated using 162 nM PMA for 24 h. For undifferentiated 3T3-L1, cells plated at 35,000 cells per well and allowed to grow for 2 days in DMEM + 3% HI FBS. 3T3-L1 adipocytes were differentiated as previously stated. Stationary phase (day 9 post-inoculation) *Coxiella burnetii* cultures were pelleted (4,000 rpm, 15 min, 4C), resuspended in high-glucose DMEM (Gibco) supplemented with 5% heat inactivated fetal bovine serum (% vol/vol), and sonicated for 15 min (VWR Aquasonic 50T) prior to addition to cells. All cells were infected with a multiplicity of infeciton (MOI) of 20 for ~6 h in the appropriate media. Bacteria was removed, and appropriate media was replaced. Adipocyte media changed every 2 days. Luminescence readings were taken after media changes. Atglistatin experiments used 20 µM atglistatin (Cayman Chemical 15284) as previously described (44). For luminescence growth curves, all cells used media without phenol red, and luminescence was measured using the Tecan Infinite M1000 plate reader (Tecan, Mannedorf, Switzerland).

For *C. burnetii* GE enumeration (at indicated time points), cells were collected, pelleted (4,000 rpm, 15 min, 4C), and snap frozen, and kept at −20°C until ready for further processing. All samples were processed using the Qiagen DNeasy Blood and Tissue Kit according to manufacturer’s instructions. GEs were quantified, and fold replication was determined by dividing the number of GEs at the indicated time point by the number of GEs at 6 h post-infection (day 0). All growth experiment data are representative of at least two technical replicates and three biological replicates.

### Fluorescence microscopy

Cells were plated (200,000 cells per well in 24-well plate) on cover slips treated with 0.5 mg/mL poly-L-lysine (Sigma P1274). Cells were washed with PBS and fixed in 4% paraformaldehyde (PFA) for 20 min on ice. Cells were permeabilized with 0.5% bovine serum albumin (BSA) (Sigma A9647), 0.2% saponin (Sigma 84510), and 0.5% HI FBS in PBS for 30 min. Antibody solutions were all prepared in 1% BSA and 0.3% saponin in PBS. Samples were incubated with primary antibodies at the concentrations indicated: mouse anti-human LAMP-1 H4A3-C (Developmental Studies Hybridoma Bank) (1:500), polyclonal rabbit anti-*C*. *burnetii* (1:2,000), rat anti-mouse LAMP-1 clone 1D4B (Thermo) (1:100). Samples were then incubated with secondary antibodies at 1:5,000 dilutions: goat anti-mouse 546, goat anti-rat 568, goat anti-rabbit 647 (Alexa Fluor 546, 568, 647; Invitrogen). BODIPY 493/503 (Cayman Chemical 25892) was added to cells at 2 µM for 20 min. Cells were mounted using Prolong Glass Antifade Mountant (Invitrogen P36984) with 1 µg/mL Hoechst 33342 (Thermo). Images were acquired using a Nikon Eclipse TE2000-S inverted fluorescence microscope equipped with a Nikon 100×/1.4 numerical aperture objective lens. Images were analyzed in SlideBook software (Intelligent Imaging Innovations). All micrographs are representative of at least two technical replicates and three biological replicates.

### Mouse infection and *in vivo* imaging

A/J, C57BL/6J, B6.129S4-C3^tm1Crr^/J, or NOD.Cg-Prkdcscid/J mice (female, 6 weeks old) were purchased from Jackson Laboratories and housed in sterile microisolator cages in the animal biosafety level 2 environment. Mice were given free access to autoclaved nutritional pellets and water and autoclaved tissue pads for enrichment; mice were monitored daily by investigators, and cage maintenance was performed by Yale Animal Resources Center. Mice with black fur had the ventral fur removed prior to infection; hair removal was achieved through a combination of shaving and brief Nair treatment until skin was smooth. Mice were infected with 1 × 10^9^ *C. burnetii* GEs suspended in 100 µL sterile PBS via the IP route. For intranasal infections, A/J mice were anesthetized with 100 mg/kg of ketamine and 10 mg/kg of xylazine in PBS via intraperitoneal injection and intranasally infected with 1 × 10^9^ bacteria in 40 µL PBS (given in 10 µL increments using a pipet in alternating nostrils until all inocula were administered). For immunosuppression, mice were treated intraperitoneally with 5 mg/kg dexamethasone (Sigma D9184) in PBS; halfway through treatment, it was switched to dexamethasone sodium phosphate (Sigma 1177032). For *in vivo* imaging, mice were anesthetized using isoflurane (Covetrus 11695067772) in an induction chamber (2 Lpm) using the XGI-8 anesthesia system. Mice were transferred to the IVIS Spectrum Instrument (Perkin Elmer) and placed in a nose cone manifold to maintain isoflurane anesthesia (0.25 Lpm). Whole animals were imaged daily using auto exposure settings (maximum of 1 min exposure for intraperitoneal infection and 5 min for intranasal infections). Mice with greater than 25% weight loss from their initial starting weight who did not begin recovery within 24 h were considered moribund and euthanized. All mouse experiments were performed with three to five mice per group.

### Tissue collection and processing

Mice were anesthetized using isoflurane as described above. Serum samples were collected via retro-orbital bleeds or terminal cardiac puncture. Retro-orbital bleeds were done under anesthesia using 100 µL capillary tubes (VWR). Terminal cardiac punctures were done under anesthesia using a 7/8” 25G needle. Cervical dislocation was used as secondary euthanasia.

Collected blood was allowed to clot for 15–30 min at room temperature, then placed on ice until ready for further processing. Blood samples were centrifuged (1,000 *g*, 10 min, 4C) and serum was separated from red blood cells via pipet. For cytokine analysis, serum samples were diluted 1:1 in PBS and filter sterilized using 0.22 µm MilliporeSigma Ultrafree-MC Centrifugal Filter Devices with Durapore Membrane (Fisher UFC30GV0S). Cytokine multiplex analysis was done by Eve Technologies using the Mouse Focused 10-Plex Discovery Assay in the Luminex 200 platform (68).

Spleens, livers, and abdominal adipose tissue were removed and imaged individually using the IVIS Spectrum Instrument on auto exposure settings. Tissues were placed on ice in sterile PBS until further processing. A small piece from each tissue sample was taken for GE analysis using the Qiagen DNeasy Blood and Tissue Kit according to manufacturer’s instructions. GEs were quantified; when appropriate, normalization to PBS control mice was done by subtracting the average of the background signal detected in PBS control organs from each sample. Excess tissue was stored at −80°C.

### Analysis of adipocytes *in vivo*

Visceral adipose tissue was collected as described above, cut into pieces using scissors, and placed in 5 mL PBS in a 50 mL conical tube. Samples were kept on ice until further processing. Adipose was treated with Liberase TL (0.1 mg/mL, Sigma-Aldrich 5401020001) at 37°C for 60 min with gentle mixing every 5 min. Red blood cells were lysed using 1× red blood cell lysis buffer at 4°C for 5 min (BioLegend 420301). Samples were centrifuged at 300 *g* for 4 min at 4°C. After centrifugation, adipocytes (which float) were separated from the SVF using a 5 mL pipet and were placed in a separate 50 mL conical tube; supernatant from the SVF was aspirated. All cells were resuspended in 5 mL PBS. Cells were centrifuged again; SVF supernatant was aspirated, and adipocyte subnatant was removed using a 1 mL pipet. All cell fractions were resuspended in 1 mL PBS and placed into a clear 24-well plate (samples were separated within the plate to ensure the luminescent signal from one sample did not bleed into another). Cell viability and separation were confirmed using a hemocytometer and Trypan blue 0.4% (Gibco 15250061). Plates were imaged using the IVIS Spectrum Instrument (Perkin Elmer) using auto-exposure settings and 1 min maximum exposure.

### Figures and statistical analysis

All statistical analyses were performed using GraphPad Prism software (GraphPad Software). All statistical tests are noted in figure legends, and only significant values are labeled.

Animal group numbers were based on previous studies and ranged from three to five mice per group (21, 22). Mice with non-productive infections due to receiving an intradermal injection were removed from analyses. This was defined as mice whose initial inoculation remained contained to the injection site (did not spread throughout the peritoneal cavity) and did not display gross symptoms of disease including weight loss, scruffy fur, hunched posture, or lethargy. All figures were created using BioRender.

## SUPPLEMENTAL MATERIAL

Fig. S1 iai.00080-25-s0001.tif Infections of SCID mice with *C. burnetii* Nine Mile phase II.

Fig. S2 iai.00080-25-s0002.tif Intranasal infections with bioluminescent *C. burnetii* strains.

Fig. S3 iai.00080-25-s0003.tif *C. burnetii* luminescence is inversely correlated with SCV formation.

Fig. S4 iai.00080-25-s0004.tif *Ex vivo* BLI imaging identifies tissues colonized by *C. burnetii*.

Fig. S5 iai.00080-25-s0005.tif Dexamethasone-mediated immunosuppression of *C. burnetii* NMII-infected A/J mice.

Fig. S6 iai.00080-25-s0006.tif *C. burnetii* NMII replication in cells treated with atglistatin.

Supplemental material iai.00080-25-s0007.docx Supplemental figure legends.

ASM does not own the copyrights to Supplemental Material that may be linked to, or accessed through, an article. The authors have granted ASM a non-exclusive, world-wide license to publish the Supplemental Material files. Please contact the corresponding author directly for reuse.
