# Supplementary figures and images for: Bioluminescent imaging to investigate Coxiella burnetii pathogenesis identifies adipose tissue as a host niche for infection
Source: Infect Immun. 2025 Jun 30;93(8):e00080-25. doi: 10.1128/iai.00080-25 (PMC12341371; doi:10.1128/iai.00080-25)

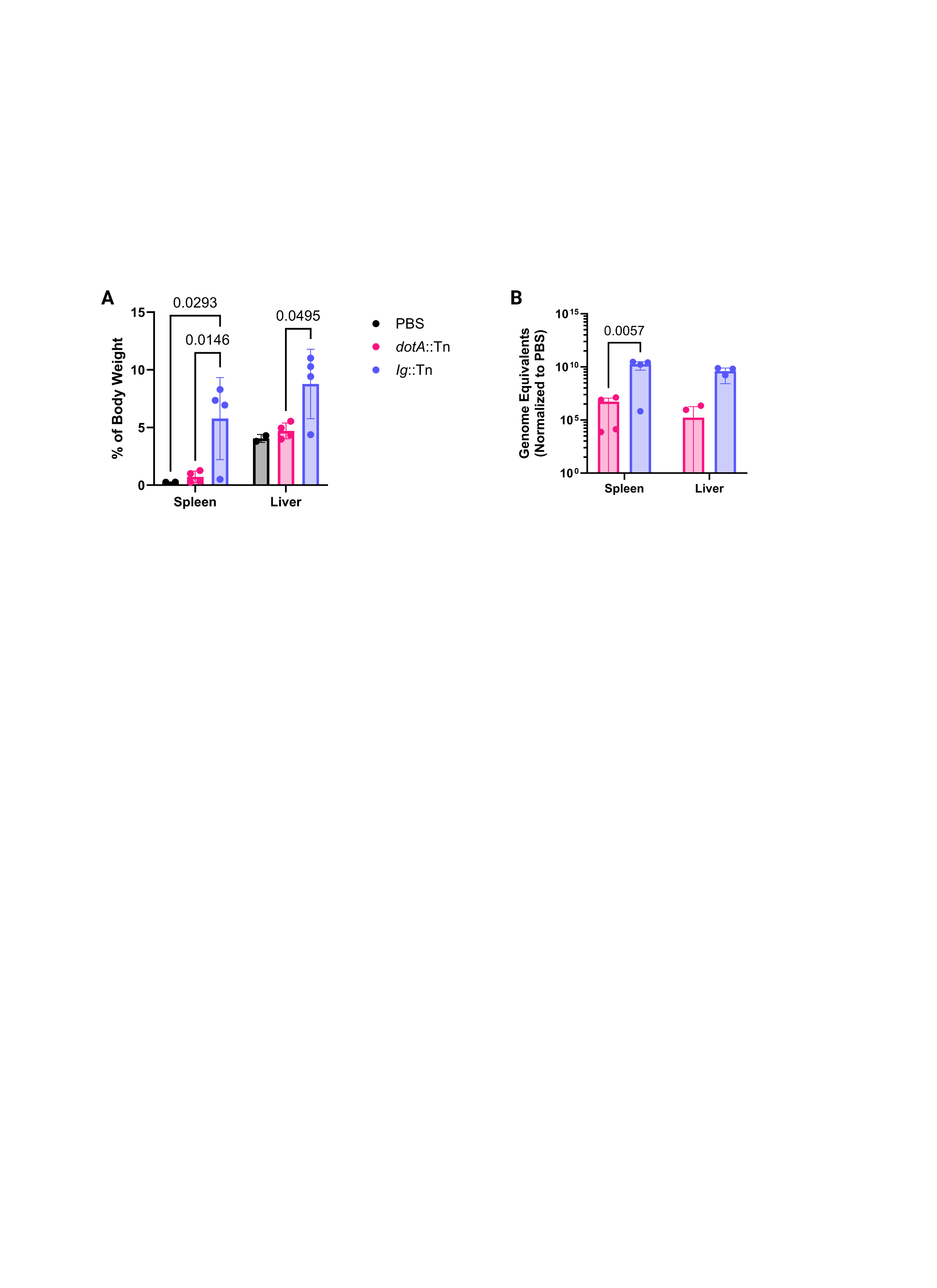

Supplement: Fig. S1 — Infections of SCID mice with C. burnetii Nine Mile phase II. [file iai.00080-25-s0001.tif]

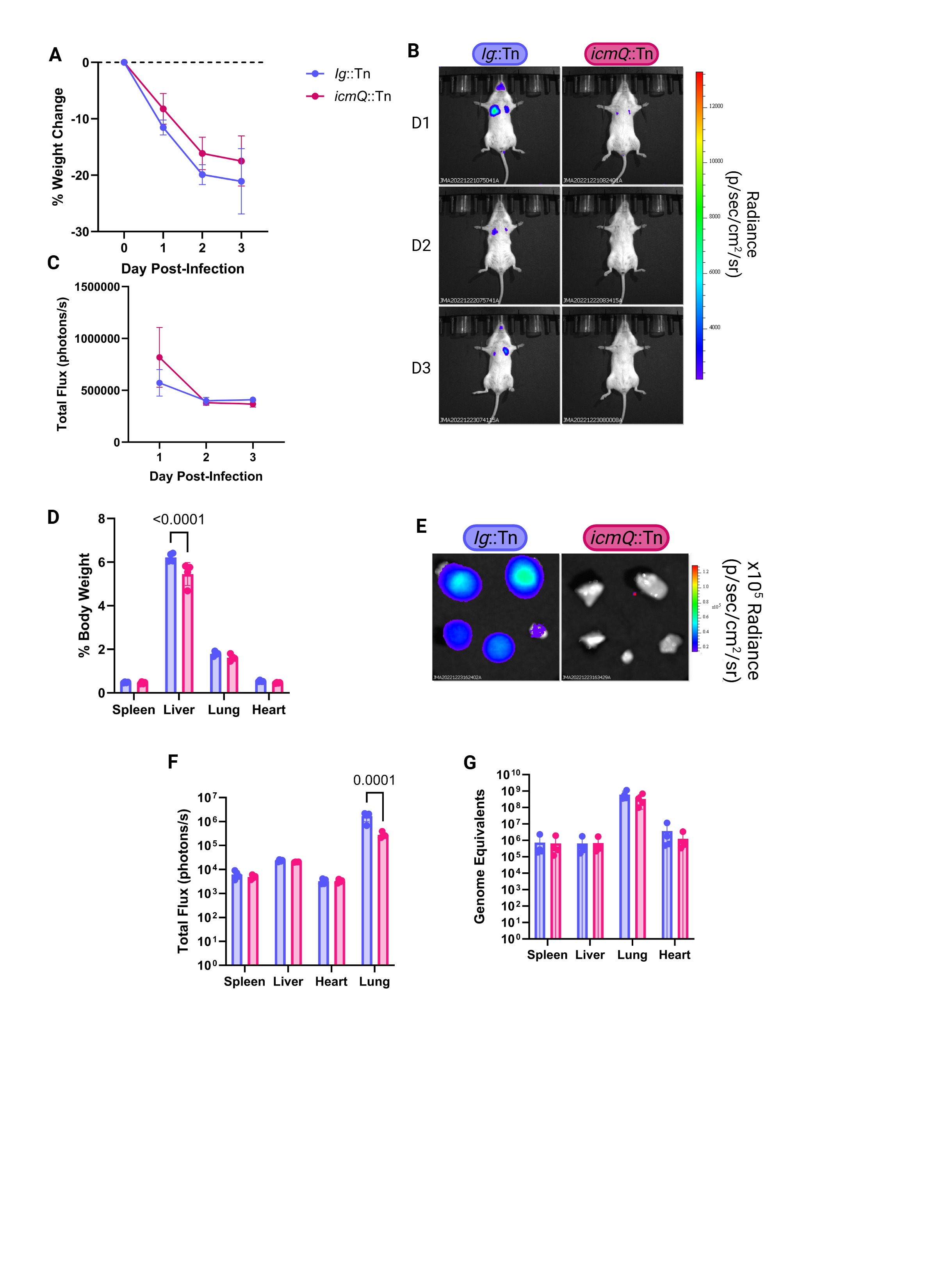

Supplement: Fig. S2 — Intranasal infections with bioluminescent C. burnetii strains. [file iai.00080-25-s0002.tif]

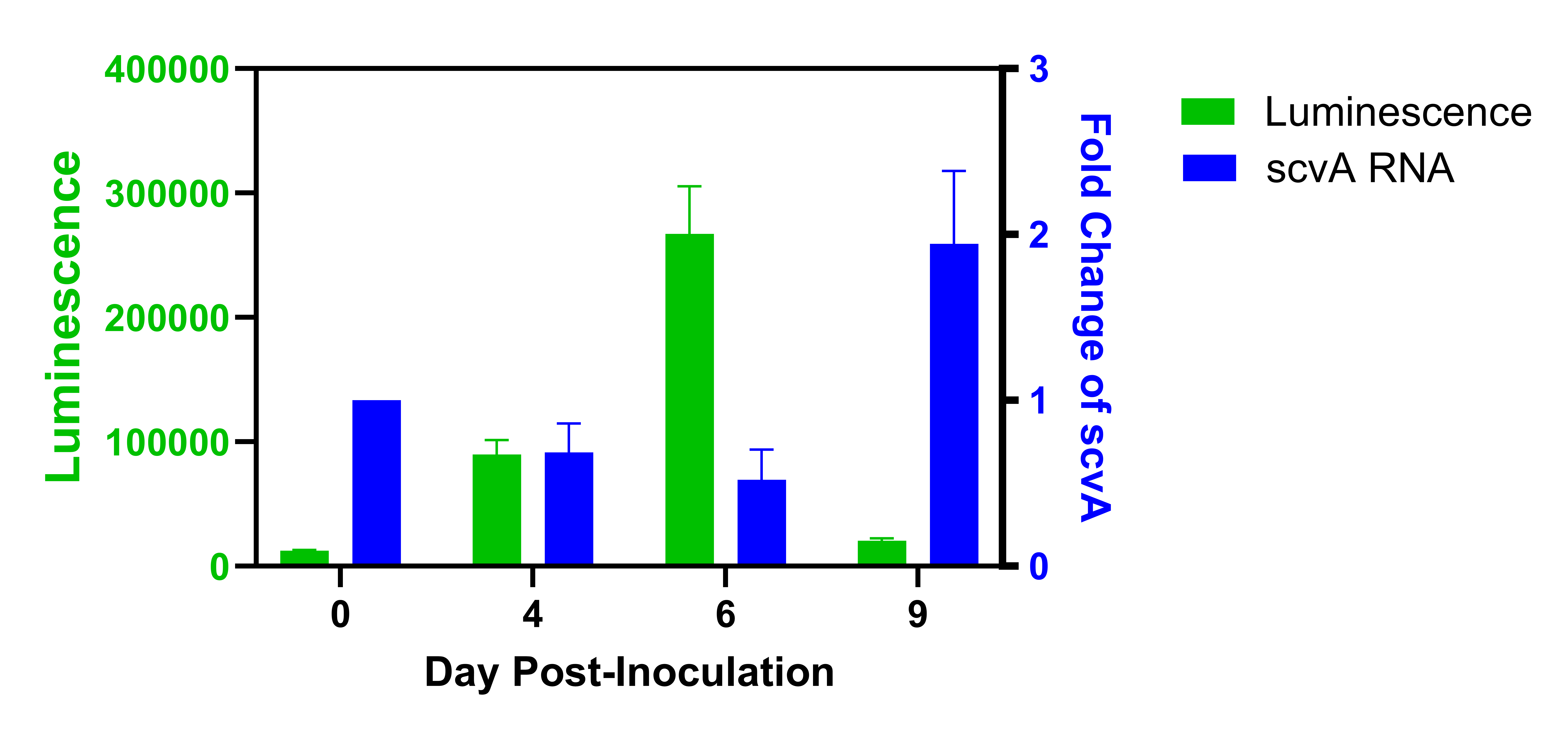

Supplement: Fig. S3 — C. burnetii luminescence is inversely correlated with SCV formation. [file iai.00080-25-s0003.tif]

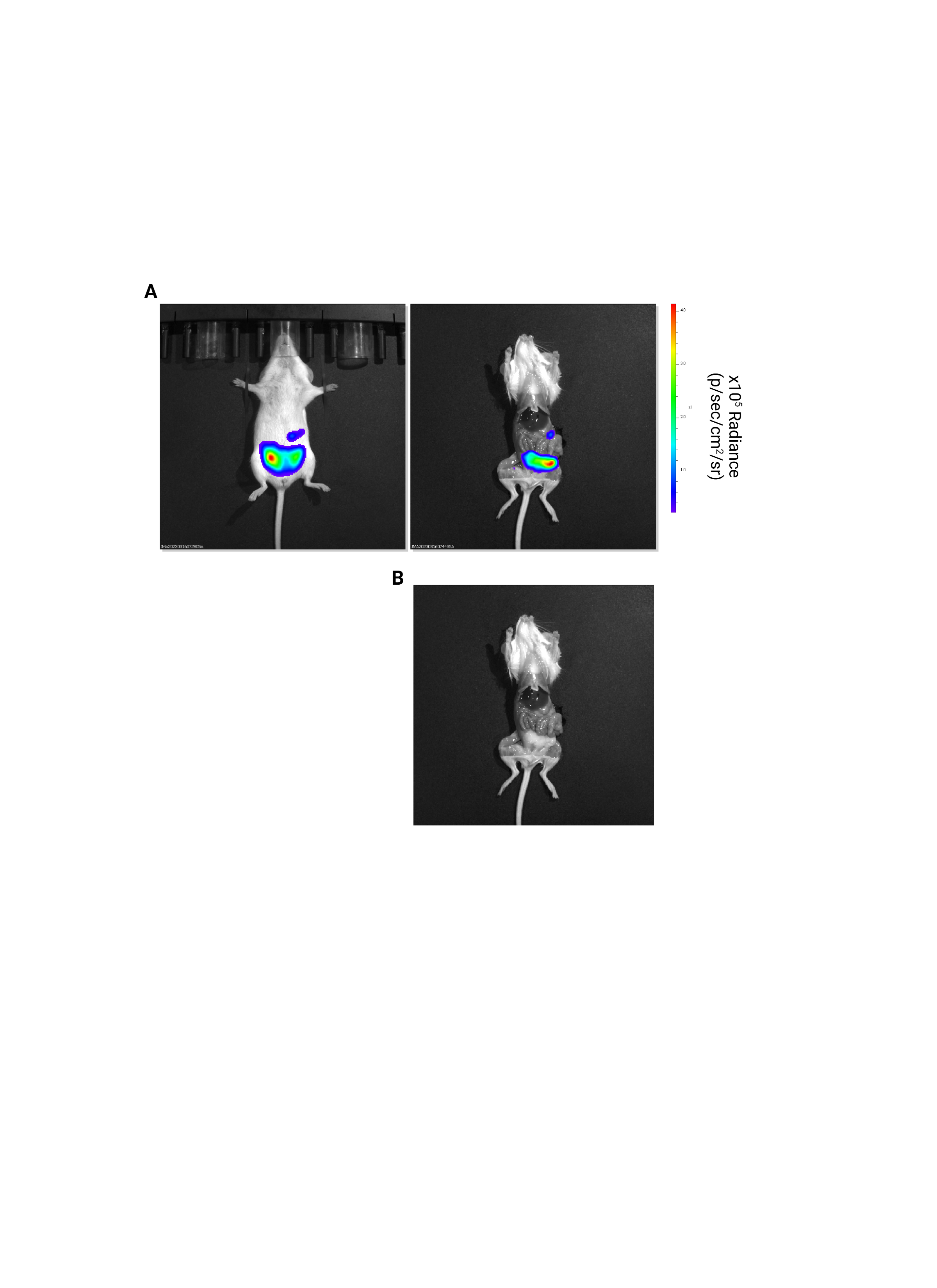

Supplement: Fig. S4 — Ex vivo BLI imaging identifies tissues colonized by C. burnetii. [file iai.00080-25-s0004.tif]

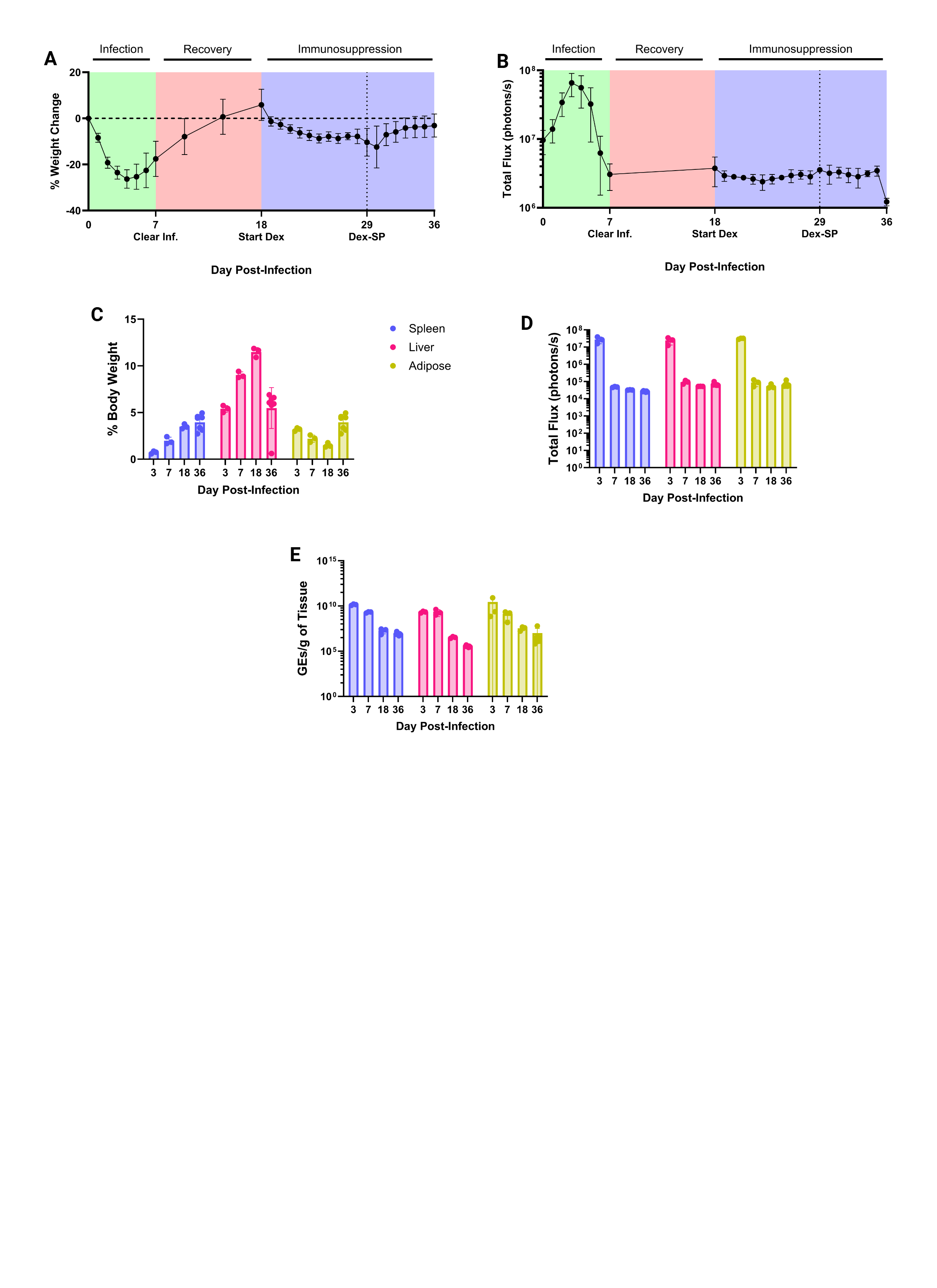

Supplement: Fig. S5 — Dexamethasone-mediated immunosuppression of C. burnetii NMII-infected A/J mice. [file iai.00080-25-s0005.tif]

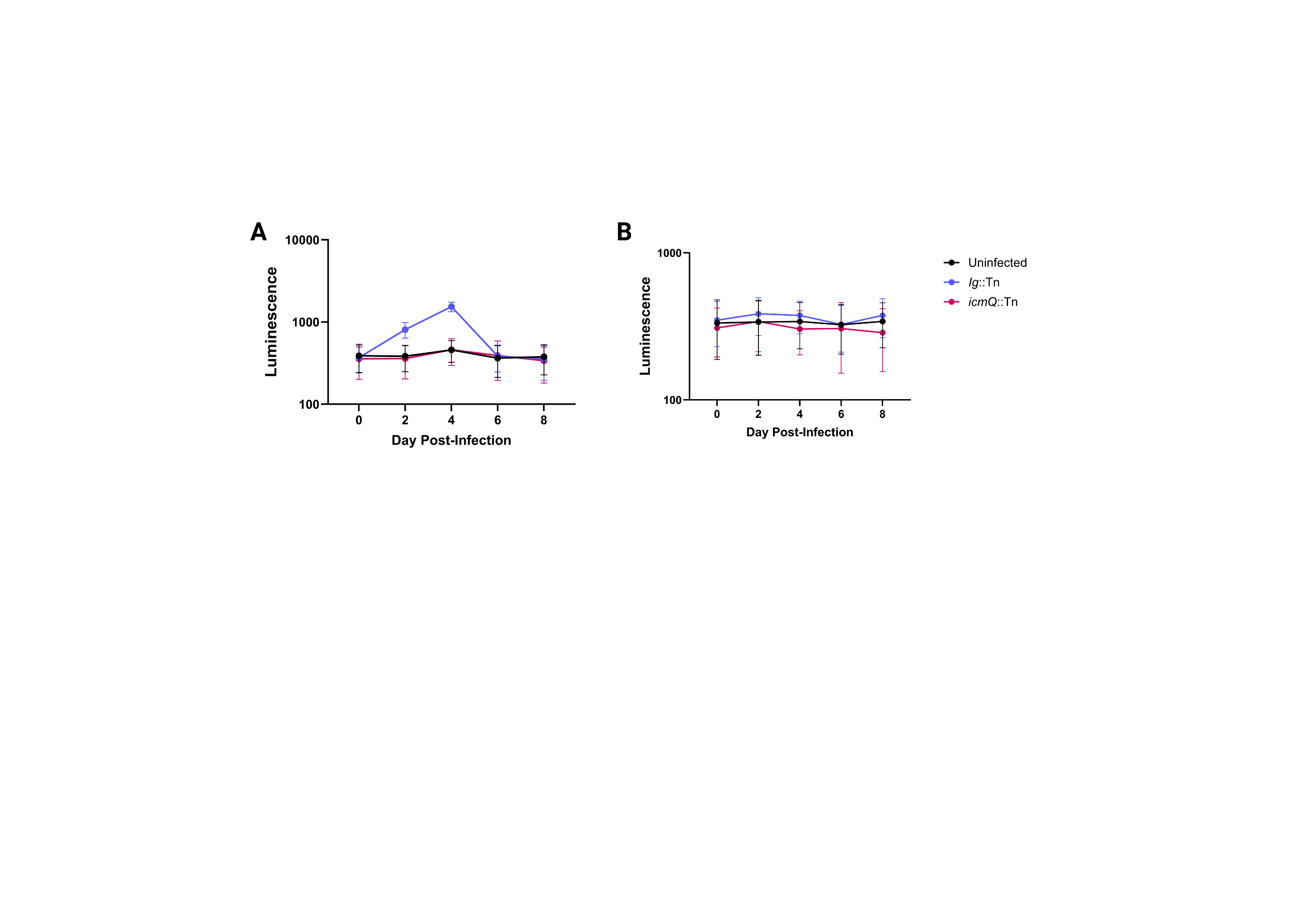

Supplement: Fig. S6 — C. burnetii NMII replication in cells treated with atglistatin. [file iai.00080-25-s0006.tif]
