# Supplementary material for: Bioluminescent imaging to investigate Coxiella burnetii pathogenesis identifies adipose tissue as a host niche for infection
Source: Infect Immun. 2025 Jun 30;93(8):e00080-25. doi: 10.1128/iai.00080-25 (PMC12341371; doi:10.1128/iai.00080-25)
Supplement: Supplemental material — Supplemental figure legends. [file iai.00080-25-s0007.docx]

**Supplemental Figure 1: Infections of SCID mice with *C. burnetii* Nine Mile phase II.** **A)** Hepatosplenomegaly on day 10 post-infection in SCID mice infected with indicated non-luminescent *C. burnetii* strains shown as organ weight as a percentage of total body weight. P-values based on two-way ANOVA and Tukey’s multiple comparison’s test with a single pooled variance are indicated. **B)** *C. burnetii* genome equivalents (GEs) on day 10 post-infection with indicated strains in spleens and livers normalized to PBS controls. Significant p-values (p<0.05) based on two-way ANOVA and Sidak’s multiple comparison’s test with a single pooled variance are indicated. Data representative of 4 technical replicates, and two biological replicates.

**Supplemental Figure 2: Intranasal infections with bioluminescent *C. burnetii* strains.** **A)** Percent change in body weight of NMII-infected mice. **B)** Representative BLI images of mice infected with indicated luminescent NMII strains at different days post-infection (D1-D3). Radiance color scale: Min = 2.17e^3^, Max = 1.33e^4^. **C)** Total flux (photons/sec) of infected mice. **D)** Organ weight as a percentage of total body weight on day 3 post-infection. P-values based on two-way ANOVA and Tukey’s multiple comparisons test with a single pooled variance, are indicated. **E)** Representative BLI images of isolated lungs of infected mice on day 3 post-infection. Radiance color scale: Min = 1.55e^4^, Max = 1.30e^5^. **F)** Total organ flux (photons/sec) on day 3 post-infection. P-values based on two-way ANOVA and Tukey’s multiple comparisons test with a single pooled variance, are indicated. **G)** Genome equivalents (GEs) extracted from organs on day 3 post-infection. Significant p-values (p<0.05) based on two-way ANOVA and Tukey’s multiple comparisons test with a single pooled variance, are indicated. Data representative of 4 technical replicates, and one biological replicate.

**Supplemental Figure 3: *C. burnetii* luminescence is inversely correlated with SCV formation.** Ig::Tn was grown axenically in ACCM-D. Luminescence and RNA levels of *scvA* were measured at the indicated time points. Fold change is *ddCq* relative to *scvA* concentrations on day 0. Data representative of three technical replicates, and three biological replicates.

**Supplemental Figure 4: *Ex vivo* BLI imaging identifies tissues colonized by *C. burnetii*.** **A)** *In vivo* and *ex vivo* BLI imaging of the same mouse infected with the Ig::Tn strain at day 3 post-infection. Radiance color scale: Min = 2.13e^4^, Max = 4.14e^5^. **B)** *Ex vivo* image without BLI overlay.

**Supplemental Figure 5: Dexamethasone-mediated immunosuppression of *C. burnetii* NMII-infected A/J mice.** All mice were intraperitoneally infected with the Ig::Tn strain and allowed to recover to their starting weight before starting immunosuppression. Immunosuppression was accomplished through daily injections of 5 mg/kg dexamethasone in 100ul PBS. Dex-SP refers to Dexamethasone sodium phosphate. **A)** Percent change in body weight of mice over time. **B)** Total flux (photons/sec) of mice over time. **C)** Organ weight as a percentage of total body weight. **D)** Total flux (photons/sec) of organs over time. **E)** GEs/g of tissue for each organ over time. Data representative of 3 technical replicates, and one biological replicate.

**Supplemental Figure 6: *C. burnetii* NMII replication in cells treated with atglistatin.** A) NMII replication in PMA differentiated THP-1 macrophages treated with 20uM atglistatin. B) NMII replication in 3T3-L1 adipocytes treated with 20uM atglistatin. Data gathered in the same experiment as those in Figure 5 A and B. Data representative of two technical replicates, and two biological replicates.
